# Structure and Luminescent Properties of Glasses in the GeS_2_-Ga_2_S_3_-Sb_2_S_3_:Pr^3+^ System

**DOI:** 10.3390/ma16134672

**Published:** 2023-06-28

**Authors:** Andrey Tverjanovich, Yurii S. Tveryanovich, Christina Shahbazova

**Affiliations:** Institute of Chemistry, St. Petersburg State University, 198504 St. Petersburg, Russia; tys@bk.ru (Y.S.T.); hristka11@yandex.ru (C.S.)

**Keywords:** chalcogenide glasses, luminescent materials, IR materials

## Abstract

The physicochemical, optical, and luminescent properties and structures of glasses of the Ga_2_S_3_-GeS_2_-Sb_2_S_3_:Pr system have been studied in a wide range of concentrations of the main components in order to reveal their correlation with the composition. According to the calculations using the Judd–Ofelt theory, glasses with a high content of Sb_2_S_3_ should provide the highest luminescence efficiency of Pr^3+^ ions. However, this result is leveled by enhancing the concentration quenching effect, followed by an increase of the Sb_2_S_3_ content in the glasses. The introduction of Pr leads to a significant increase in the fraction of Sb-Sb, Sb-Ge, Ge-Ge bonds in glasses enriched with Sb_2_S_3_ and GeS_2_. In the cases of the glasses enriched with Ga_2_S_3_, this effect was not observed, apparently because Ga promotes the formation of three-coordinated sulfur atoms.

## 1. Introduction

Chalcogenide glasses—due to a high transparency in a wide spectral range of 0.5–20 µm, low optical losses in the mid-infrared region, high refractive index varied from 2 to 3.3, and a high coefficient of optical nonlinearity—have a great advantage as optical materials, including fiber and planar optics [1,2].

Chalcogenide glasses are of interest not only as passive optical materials, but also as active luminescent media for the IR range if doped with rare earth ions (REIs). These materials are especially interesting for the development of optically active fibers suitable for the IR region [2,3]. This is due to the rather large optical losses in chalcogenide fibers (the best value is 12 dB/km in a multimode As_2_S_3_ fiber) [4], and, accordingly, the need for amplification when transmitting a signal over relatively long distances [5].

Taking into account the characteristic absorption of organic groups in the mid-IR range, chalcogenide glasses doped with REIs have undeniable advantages as materials for optical sensors used in chemistry, medicine, biology, and many other fields of science and industry [2,6,7].

Despite their attractiveness, there are still a number of unresolved problems associated with their application. One of these problems is a high quenching level of the REI luminescence due to the uneven distribution of rare earth ions in the glass matrix. The latter decreases the luminescence efficiency [8].

One of the main chalcogenide systems, which was studied as a chalcogenide matrix for the introduction of REIs, is the Ga_2_S_3_-GeS_2_ quasi-binary system. The importance of this system can be associated with the formation of GaS_4/2_^−^ complex structural units, which facilitate the solubility of rare earth ions in the glass matrix [9,10]. However, it has several disadvantages, including a high synthesis temperature and a high crystallization capacity. The latter is especially critical for fabrication of optical fibers. To reduce these negative factors, the composition of the glass-forming matrix was complicated by adding a third component—Sb_2_S_3_ [11].

The structure of the two glass compositions Ge_22_Ga_3_Sb_10_S_65_ and Ge_15_Ga_10_Sb_10_S_65_ of the Ga_2_S_3_-GeS_2_-Sb_2_S_3_ system was studied by neutron diffraction, X-ray diffraction, and EXAFS. It was shown that Ge, Ga, Sb, and S coordination realized in glasses is 4, 4, 3, and 2, respectively. The Sb-S distances were found in the glass structure, which are 0.3–0.4 Å longer than the length of the covalent bond. On the basis of which, it was suggested that Sb atoms can have different local environments [12]. The glass formation region, density, refractive index, thermal expansion coefficient, T_g_ of this ternary system were studied in [13]. The additivity of the density and refractive index was shown.

In general, there are various REIs, which were introduced into chalcogenide glasses. These are mainly Er, Nd, Pr, Dy Ho. In the current work, Pr^3+^ was chosen as an REI, because it has not only luminescence properties at one of the wavelengths of information communication lines (1.3 μm), but it also exhibits a broadband luminescence in the mid-IR region of the spectrum between 3.5 and 5.2 μm, suitable for the fabrication of detectors sensitive to CO_2_, CO, and N_2_O [14,15]. In addition, luminescent glasses of this system containing Er^3+^ [11,16,17], Ho^3+^ [18], Nd^3+^, and Dy^3+^ [19] ions were previously studied, but we did not find any information on studies of glasses of this ternary system containing Pr^3+^ ions. So far, only glasses with selenium instead of sulfur [20] or glasses belonging to quasi-binary sections were studied. Thus, this study is aimed at the investigation of the physicochemical, optical, and luminescent properties and structures of glasses of the Ga_2_S_3_-GeS_2_-Sb_2_S_3_:Pr pseudo-ternary system in a wide range of concentrations of the main components in order to reveal their correlation with the composition. Moreover, it is necessary to reveal the correlation between the chemical structure of the glass matrix and the uniformity distribution of Pr^3+^ ions in it.

According to the studies carried out, glasses of this system with a high content of Sb_2_S_3_ have a high crystallization stability. In turn, due to the Judd–Ofelt theory, they should have a high efficiency of Pr^3+^ luminescence. At the same time, these glasses are characterized by a high degree of inhomogeneity in the distribution of Pr^3+^ ions in the glass matrix, which leads to strong concentration quenching. The effectiveness of increasing the content of Ga_2_S_3_ in glass as an agent promoting the dissolution of Pr in the glass matrix is highly limited in concentration. The nature of the concentration changes in the properties of glasses at a high content of Ga_2_S_3_ and Sb_2_S_3_ is not monotonic, indicating some kind of structural–chemical interaction of the corresponding structural units in the glass. The introduction of Pr into glasses dramatically changes the distribution of chemical bonds in them, increasing the proportion of metal–metal (M-M) bonds.

## 2. Materials and Methods

### 2.1. Materials

**Figure 1:**
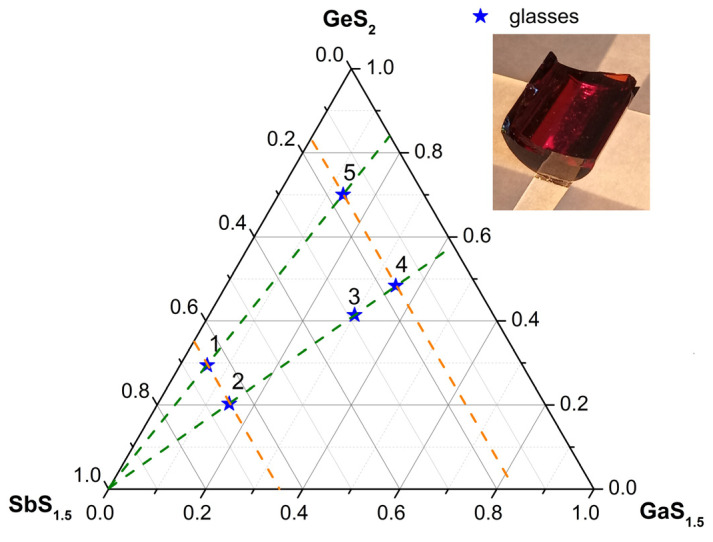
The studied compositions (asterisks, numbers) and the sections to which they belong (dotted lines) in the GeS_2_-GaS_1.5_-SbS_1.5_ ternary diagram. A photo of the glass composition **4** is shown in the inset.

The glass compositions for the synthesis and study were chosen so that they belonged to four cuts on the concentration triangle (GeS_2_-GaS_1.5_-SbS_1.5_) (Figure 1, Table 1). In the first group of cuts, at a fixed relative content of Sb_2_S_3_, the mutual ratio of Ga_2_S_3_ and GeS_2_ changed (two sections). In the second group of cuts, at a fixed ratio of Ga_2_S_3_ and GeS_2_, the relative content of Sb_2_S_3_ changed (two sections). These choices of compositions make it possible to study the influence of each of the components on the properties of the chosen glasses. In addition, the literature data on the region of glass formation in this system were also taken into account when choosing the aforementioned composition [16]. The selected compositions are marked on the concentration triangle in Figure 1 with an asterisk and a serial number.

The glass synthesis was carried out by fusing pure components in evacuated to high vacuum and sealed quartz ampoules during several stages in a constantly swinging furnace. The maximum synthesis temperature was 950 °C. All glasses were obtained by quenching a quartz ampoule with a sample melt from the synthesis temperature in air. Thus, glassy matrices were synthesized for the further preparation of glasses containing praseodymium ions with different concentrations. The synthesis of glasses with praseodymium was carried out in one stage using a similar procedure.

### 2.2. Methods

For the resulting glasses, the density was measured by hydrostatic weighing in toluene. The refractive index was measured by the change in the focal length in an IR microscope at a wavelength of 1.2 μm after the introduction of a plane–parallel sample of known thickness between the objective and the focus. A differential thermal analysis (DTA) was carried out during heating at a constant rate of 10 K/min.

X-ray diffraction studies of the samples were carried out on an automatic powder diffractometer D2 Phaser (Bruker, Billerica, MA, USA) with the following parameters: CuKα_1+2_ X-ray tube radiation, sample rotation speed 20 rpm, diffraction angle interval 2 theta = 7–60°, scanning step of 0.02°.

Optical absorption spectra were measured on plane–parallel polished samples. The measurements were performed on a UV-3600 spectrometer (Shimadzu, Kyoto, Japan) in the spectral range between 0.5 and 3.2 µm and on a Tensor 27 spectrophotometer (Bruker, USA) in the spectral range between 2.5 and 25 µm.

The Raman spectra of glassy samples were measured on a Senterra Raman spectrometer (Bruker, USA) with an Olimpus BX-52 optical microscope at room temperature. A 785-nm laser was used as the excitation source. The laser power was reduced to 1 mW to prevent sample heating.

Luminescence spectra were measured on a Fluorolog-3 spectrofluorimeter (Horiba Jobin Yvon, Takamatsu, Japan). The beam of the pump laser was aimed at the sample surface at an angle of approximately 90 degrees. At an angle of ~45 °C, a light guide was brought to this surface at a distance of ~2 cm from the surface in such a way that the reflected beam of the pump laser did not fall into the light guide. The luminescence excitation spectrum was measured at a wavelength of 1040 nm to determine the most effective excitation wavelength. Subsequently, the value of the pump wavelength determined in this way (607 nm) was used to excite luminescence.

The calculation of the radiative lifetime, transition probability, and branching coefficients, in accordance with the Judd–Ofelt theory, from the optical absorption spectra was carried out on the basis of work by Walsh [21]. The matrix elements for the calculation were taken from Reference [22].

## 3. Results and Discussion

### 3.1. Physicochemical Properties

All samples, both the matrices themselves, and the samples containing Pr^3+^ ions, were obtained in the glassy state by cooling the ampoule with the melt in air. The glassy state of the samples was monitored visually under a microscope and using XRD analysis (Figure S1a,b in Supplementary Materials (SM)). For samples **1** and **2**, visual control was done using an IR microscope because of the shift of their fundamental absorption edge to a longer wavelength region relative to the visible range.

The values of density, refractive index, T_g_, and T_cr_ for glassy matrices are given in Table 1. The obtained values of T_g_ and refractive index were consistent with the data discussed in Reference [13]. The density values were subsequently used to estimate the specific concentration of Pr^3+^ ions when calculating their absorption cross section.

The density and refractive index of glasses increased with an increased in the content of Sb_2_S_3_, while the value of T_g_ decreased, which was due to a decrease in the dimensionality of the structure due to an increase in the concentration of trigonal structural units relative to tetrahedral ones. The crystallization temperature behaved similarly. A change in the ratio of Ga_2_S_3_ and GeS_2_ at a constant Sb_2_S_3_ content did not have such an unambiguous effect on the given parameters. The effect depended on the content of Sb_2_S_3_, which may have indicated some kind of interaction between Ga_2_S_3_ and Sb_2_S_3_. This is also supported by the optical properties discussed below.

It should be noted that glasses of compositions **1** and **2** do not crystallize during heating at a rate of 10 K/min. Thus, they can be considered as promising materials for the production of bulk optical elements using rapidly developing molding technique [23].

### 3.2. Optical Properties

**Figure 2:**
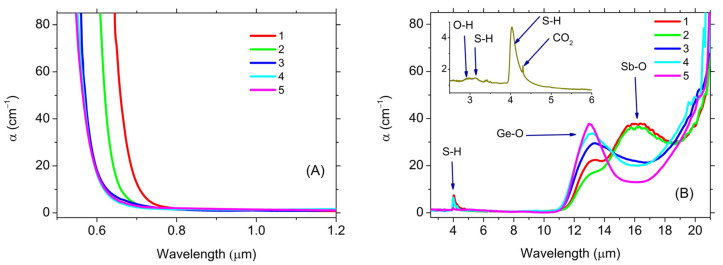
Optical absorption spectra of the studied glassy matrices. (**A**) Visible and NIR spectral range. (**B**) IR spectral range (inset is part of the spectrum on an enlarged scale). The numbers correspond to the glass compositions.

The fundamental absorption edge of the synthesized glassy matrices mainly lied in the visible range. However, for glasses with the maximum content of Sb_2_S_3_ (compositions **1** and **2**), the absorption edge lied at the long-wavelength edge of the visible range, as it can be seen from the optical absorption spectra (Figure 2A). This spectral position of the band gap agrees with the literature data on the band gap for amorphous Sb_2_S_3_, Ga_2_S_3_, and glassy GeS_2_. So, for films of amorphous Sb_2_S_3_, E_g_ = 2.2–2.5 eV [24,25]; for films of amorphous Ga_2_S_3_, E_g_ = 2.2–3.0 [26,27]; for glassy GeS_2_, E_g_ = 3.2 eV [28]. Interestingly enough, an increase in the content of Ga_2_S_3_ relative to GeS_2_ at a maximum content of Sb_2_S_3_ (compositions **1** and **2**) led to an increase in the band gap, even though E_g_ of Ga_2_S_3_ was smaller than E_g_ of GeS_2_. This may be an indication of an interaction between Sb_2_S_3_ and Ga_2_S_3_. There was no such shift observed for the samples with the minimum studied Sb_2_S_3_ content (compositions **4** and **5**).

According to the IR absorption spectra (Figure 2B), the glasses were transparent up to 11 μm. Further, transmission was limited by the presence of Ge-O impurity bonds, the absorption band of which was located in the region of 12.8 μm [29]. The intensity of this absorption increased with an increase in the relative concentration of GeS_2_ in glasses (Figure 2B). With a significant increase in the relative content of Sb_2_S_3_ in glasses (compositions **1** and **2**), a peak appeared in the region of 16.1 μm, which, apparently, was due to the presence of Sb-O bonds (the Raman spectrum of crystalline Sb_2_O_3_ was characterized by an intense band in the region of 647 cm^−1^ (15.5 μm) and a weak band at 592 cm^−1^ (16.9 μm)) [30]. Impurity Ga-O bonds (14.7 μm) [31] can also be presented in the glasses.

In addition, impurity absorption bands, due to the presence of S-H bonds (4.0 and 3.1 μm) [25], were observed in the transparency range. A small contribution of O-H (2.9 µm) [6] and CO_2_ (4.3 µm) [29] groups was also observed, as it could be seen in the enlarged IR spectrum (Figure 2B, inset).

**Figure 3:**
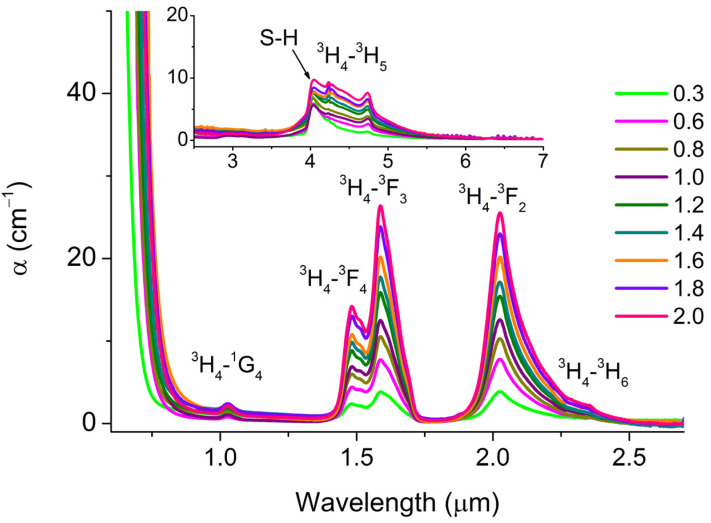
Optical absorption spectra of glasses of composition **1** with Pr content varied from 0.3 to 2 at.%.

The introduction of Pr into the glasses led to the appearance of additional absorption bands in the transparency range due to transitions between the levels of Pr^3+^ ions. The dependences of the optical absorption coefficient for glasses, for an example, of composition **1** with different Pr concentrations are shown in Figure 3. Spectral position of the absorption bands in the studied glasses, due to the transitions of Pr^3+^ ions, the energy of the transitions and their identification are given in Table S1 (Supplementary Materials). For **1** and, to a lesser extent, **2**, the ^3^H_4_-^1^D_2_ transition was overlapped by the fundamental absorption edge.

The fundamental absorption edge shifted to a longer wavelength spectral range when a REI was introduced into glass. Broadband absorption in the region of 4.74 μm is referred to as the ^3^H_4_-^3^H_5_ transition (Figure 3, inset) and was partially overlapped by an S-H impurity absorption. Therefore, the practical use of this promising transition for broadband mid-IR luminescence is possible only after an additional purification of glasses from the impurities of S-H groups—e.g., by use of getters or by synthesis from volatile halides [4].

### 3.3. Luminescent Properties

**Figure 4:**
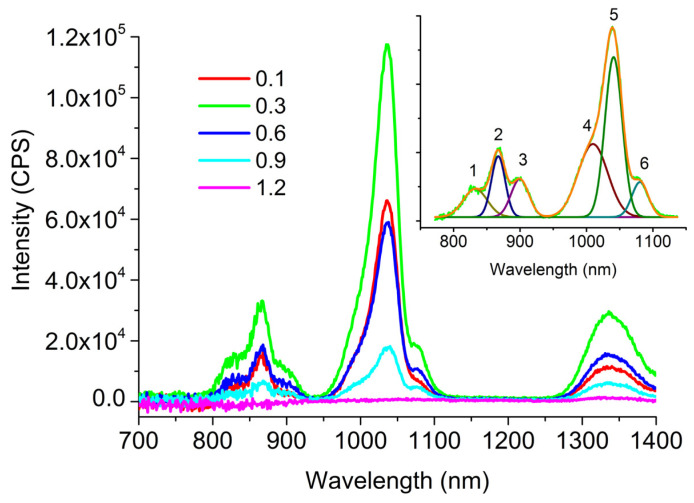
Luminescence spectra for glasses of composition **3** with different contents of Pr^3+^ ions (0.1, 0.3, 0.6, 0.9 and 1.2 at. %). The corresponding curves are marked with different line colors. The inset shows a fragment of the spectrum for the composition with 0.3 at.% Pr^3+^ and the result of its fitting by 6 peaks.

According to the method described above, the luminescence spectra of all synthesized glasses were measured upon excitation by radiation with a wavelength of 607 nm. In order to study the influence of the glass composition on the concentration quenching of luminescence, the measurement of the luminescence intensity for glasses of the same matrix, but with a different content of REIs, was carried out under strictly identical conditions. The same principle was applied to all plane–parallel glass samples to standardize the size of the sample from which the signal was collected. The luminescence spectra, for an example, for glasses of composition **3** with different contents of Pr^3+^ ions are shown in Figure 4.

Several luminescence peaks overlapped in the wavelength range between 800 and 1100 nm. In this wavelength range, luminescence spectra could be deconvoluted in six peaks (Figure 4, inset). The results of approximation of luminescence bands and their assignments are given in Table 2.

Peaks 3 and 6 were due to the presence of neodymium impurities in praseodymium metal (see Supplementary Materials).

As it can be seen from Figure 4, the luminescence intensity for **3** decreased due to concentration quenching already at Pr^3+^ ions above 0.3 at.%.

**Figure 5:**
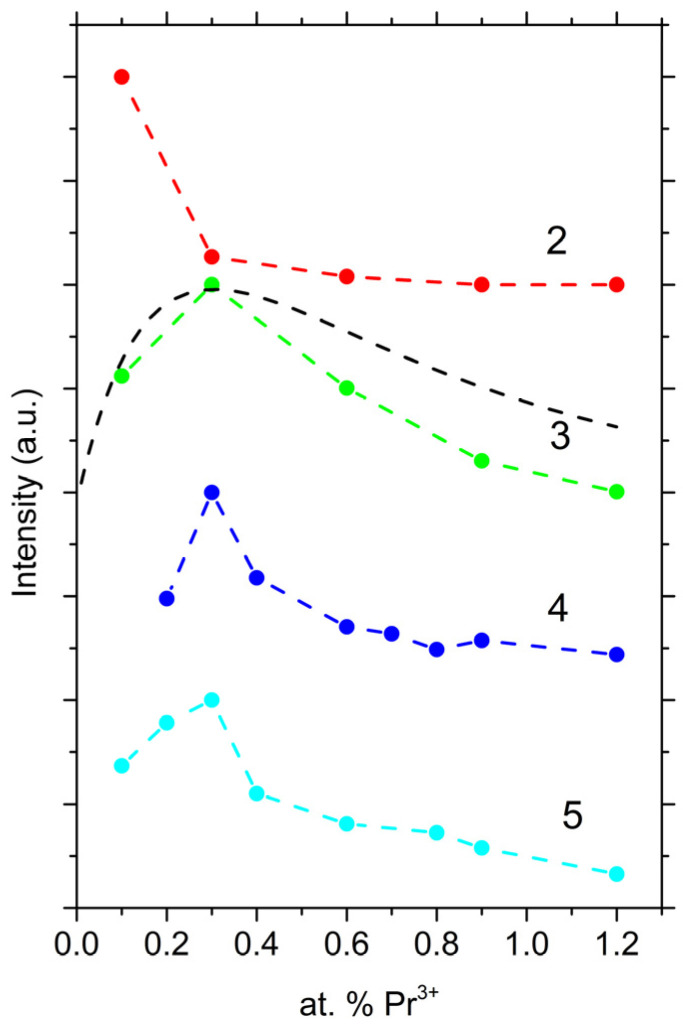
Dependence of the luminescence intensity at 1040 nm on the concentration of Pr^3+^ ions for the studied glasses (points with dashed lines). The graphs are shifted relative to each other along the y-axis for clarity. The numbers **2**–**5** correspond to the numbers of the composition. The black dotted line is an approximation according to Equations (1) and (2).

Figure 5 shows the dependences of the relative luminescence intensity at 1040 nm on the content of Pr^3+^ ions for glasses of the studied compositions.

For glasses of composition **1**, the luminescence intensity was insufficient for measurements. Possibly, this is because of the absorption of the excitation radiation by the glass matrix. For glasses of composition **2**, the maximum luminescence intensity was observed at concentrations below 0.1 at.% Pr. For glasses with a low content of Sb_2_S_3_, the maximum luminescence intensity shifted towards an increase of REI content in the glass. It should be noted that the shift of the luminescence intensity maximum toward high concentrations of Pr^3+^ ions, with an increase in the relative content of Ga, turned out to be insignificant, in contrast to glasses with Er^3+^ ions [32]. Apparently, the observed strong concentration quenching was associated with the cross-relaxation process between the ^1^D_2_ → ^3^F_4_ and ^3^H_4_ → ^1^G_4_ transitions.

Let us consider the obtained data for composition **3** from the point of view of the distribution statistics of Pr^3+^ ions in the glass matrix. The luminescence intensity (*I*) was proportional to the concentration of Pr^3+^ atoms located at a distance of more than two interatomic distances from each other. We can write the following relationship for the luminescence intensity, which reflects the nature of the distribution of Pr^3+^ ions in the glass matrix [10]:

*I ~ C⋅g*_0_ (1)

(2) $$g_{0}={\left(\frac{1-b}{1-b\left(1-S\right)}\right)}^{6}$$

where *C* is the concentration of Pr^3+^ ions and *b* is the fraction of Pr atoms in the total number of metal atoms (it is assumed that only Pr, Ge, Ga, or Sb atoms can occupy the second coordination sphere of Pr^3+^ ions). *S* is the so-called segregation factor. It is equal to 1 if the Pr^3+^ ions are distributed statistically uniformly over the metal positions of the glass structure network. On the other hand, this factor is greater than one if there is a tendency for the structural units containing these ions to agglomerate. The result of approximation for **3** is shown in Figure 5 (see black dotted line). The obtained value of the segregation factor equal to 24 indicated a very strong agglomeration and, therefore, a strong dipole–dipole interaction between Pr^3+^ ions.

### 3.4. Parameters of the Judd–Ofelt Theory for the Synthesized Glasses

For the Pr^3+^ ion, the 4f^N−1^5d band had a relative low energy and located close in energy to the ^3^P_2_, ^3^P_1_, ^1^I_6_, and ^3^P_0_ levels [33]. It contradicted the assumptions in the Judd–Ofelt theory, according to which the 4f^N−1^5d configuration should be degenerate in energy and have a significant difference in energy with the 4f^N^ configuration [34,35]. To overcome this contradiction, many different approaches have been proposed [36,37,38]. However, if we considered levels with energies less than the ^3^P_0_ level (in our case, it was even less than ^1^D_2_), then the standard method was quite sufficient for the accurate prediction of the Judd–Ofelt parameters [39]. In addition, it should be noted that all absorption bands observed and used in the calculations could be referred to the electronic transitions [40]; therefore, the magnetic dipole component could be neglected.

The calculation results are shown in Table 3 and Table 4. The values for the branching ratio are shown in Table S2 (Supplementary Materials). The standard deviation (RMS) between the theoretical and experimental line strengths was 0.15 × 10^−20^ cm^2^ in average. Such a low value indicated a very good calculation accuracy.

The obtained values of the parameters Ω_2_, Ω_4_, and Ω_6_ were consistent with the literature data for the Ge_25_Ga_5_S_70_ composition (Ω_2_ = 12.8, Ω_4_ = 4.3, Ω_6_ = 7.7) [41].

The value of the parameter Ω_2_ was associated with the degree of covalence of the bonds of the REIs and the degree of symmetry of its environment [40,42,43]. According to the data obtained, as the content of Sb_2_S_3_ and Ga_2_S_3_ in glasses increased, the degree of covalence (or the degree of asymmetry) decreased. Moreover, among the investigated glass compositions, the highest radiation probability and the lowest radiation lifetime were observed for the composition with the highest content of Sb_2_S_3_ and Ga_2_S_3_ (composition **2**). This was also consistent with the correlation of the ratio Ω_4_/Ω_6_ with the stimulated emissivity [43]. For **2**, the ratio Ω_4_/Ω_6_ was maximal. The highest calculated radiation probability was observed for the ^1^G_4_-^3^H_5_ transition, which corresponded to 1.336 μm (one of the wavelengths of information communication lines).

### 3.5. Glass Structure According to the Raman Spectroscopy Data

The GeS_2_ glass structure was composed of a GeS_4/2_ tetrahedra connected by corners or edges. In addition, there was a small proportion of non-stoichiometric Ge-Ge and S-S bonds. The Raman spectrum of GeS_2_ contained the following bands: 110, 155, 175, 209, 258, 342, 370, and 433 cm^−1^. The most intense A_1_ mode at 342 cm^−1^ was associated with completely symmetric vibration of the GeS_4/2_ tetrahedron or tetrahedra with common corners [44,45]. The 370 cm^−1^ mode was the A_1c_ companion caused by vibrations of bridging sulfur atoms in the tetrahedra connected by common edges [45]. The band at 258 cm^−1^ was referred to as a non-stoichiometric ethane-like structure S_3/2_Ge-GeS_3/2_ [46]. The band at 433 cm^−1^ could be attributed to vibrations of the edge-connected tetrahedral [45,46]. The presence of nonstoichiometric S–S bonds was associated with a mode at about 485 cm^−1^ [47]. The bands around 110 and 150 cm^−1^ were the ν_I_) and ν_4_(F_2_) vibrations of the GeS_4/2_ tetrahedron [48]. Approximation of the experimental Raman spectrum of GeS_2_ glass in the range from 280 to 470 cm^−1^ also resulted in revealing a broad peak at about 402 cm^−1^, which belonged to the F_2_ vibrations of the GeS_4/2_ tetrahedron [48,49].

Pure Ga_2_S_3_ had not been obtained in the bulk glassy state. In the multicomponent glasses containing Ga_2_S_3_, gallium was four-coordinated by sulfur, forming, like GeS_2_, a GaS_4/2_^−^ tetrahedral [50,51]. Assuming the atomic weight of Ge as similar to those of Ga, it could be expected that the frequencies of analogous vibrations were also similar. Thus, for a fully symmetrical vibration of the GaS_4/2_^−^ tetrahedral structural unit, a band was assumed at about 320 cm^−1^ [50] or 350 cm^−1^ [51]. Similarly, for Ga-Ga bonds, a band was assumed at about 260 cm^−1^ [50].

Pure Sb_2_S_3_ was also not obtained in the bulk glassy state. Films were obtained, the Raman spectrum of which was mainly characterized by one broad maximum at about 300 cm^−1^ [52]. For fully symmetric vibrations of the SbS_3/2_ pyramid in chalcogenide glasses, a band was assumed at about 290 cm^−1^ [53] and 160 cm^−1^ [54].

Thus, if the structure of glasses of the Ga_2_S_3_-Sb_2_S_3_-GeS_2_ system consisted of structural units SbS_3/2_, GaS_4/2_^−^, GeS_4/2_, then we should have observed mainly the three most intense fully symmetrical modes at 300, 320 and 340 cm^−1^, respectively. If there were non-stoichiometric Ga-Ga or Ge-Ge bonds, then a mode should appear at about 260 cm^−1^. If the GeS_2_ content dominates in glass, then Raman peaks could also be observed at about 370, 400, and 430 cm^−1^.

**Figure 6:**
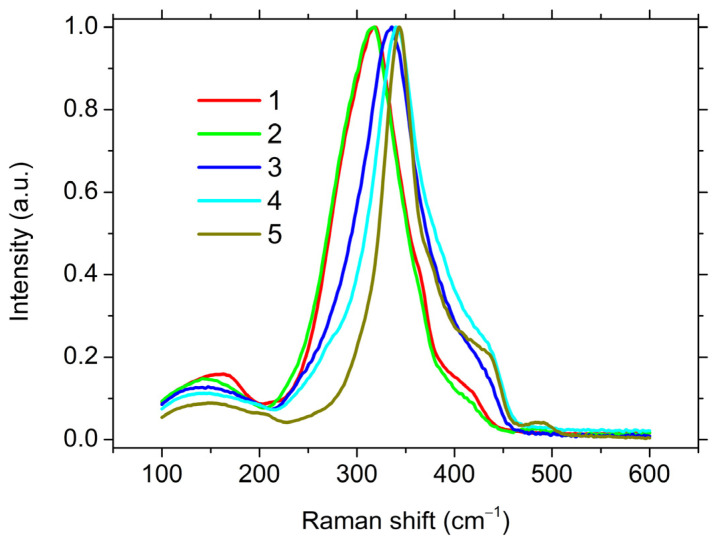
Raman spectra of the studied glassy compositions. The numbers indicate the serial numbers of the compositions.

The Raman spectra of five studied glassy matrices are shown in Figure 6. The spectra were reduced for the correction of the influence of temperature, harmonic oscillator factor, and the wavelength dependence of the scattered intensity, as was described in Reference [55].

**Figure 7:**
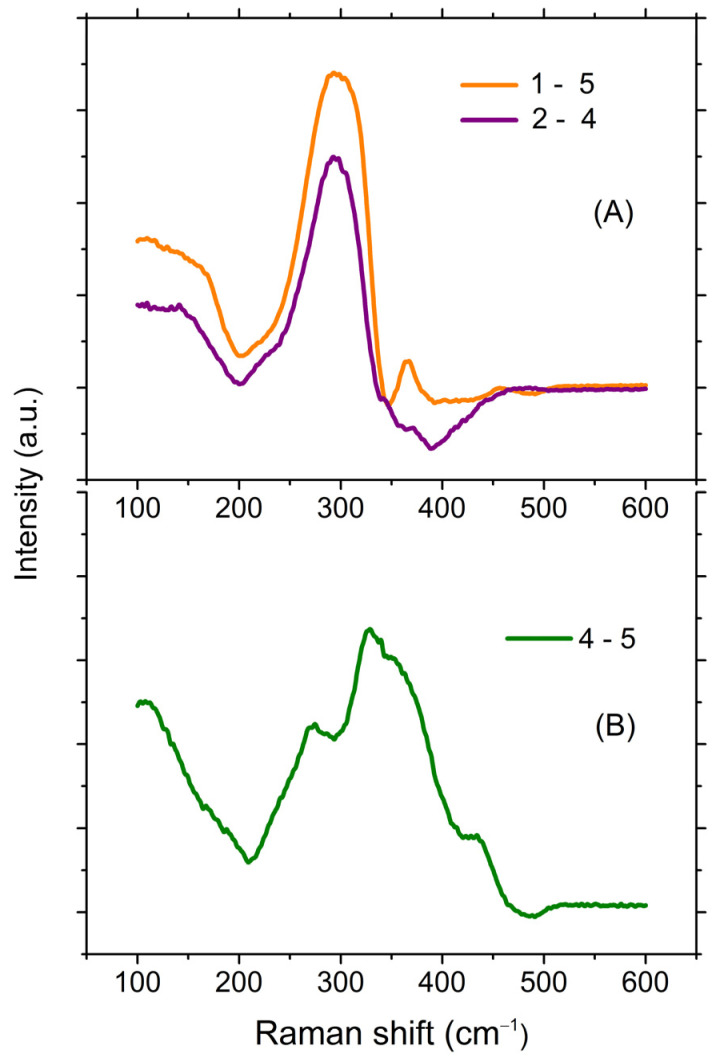
Differences in the Raman spectra of the glasses of compositions: (**A**) **1**–**5** and **2**–**4**; (**B**) **4**–**5**.

The maximum of the Raman spectrum shifts to lower frequencies and the main peak also broadened as a result of the increase in the relative content of Sb_2_S_3_ in glasses at a constant ratio of GeS_2_ to Ga_2_S_3_ (the sections marked with dark green dotted lines in Figure 1). This was consistent with the difference in vibration frequencies of GeS_4/2_ (GaS_4/2_^−^) and SbS_3/2_ structural units. At the same time, when the ratio of GeS_2_ to Ga_2_S_3_ changed at a constant content of Sb_2_S_3_ (the sections marked with orange dotted lines in Figure 1), the changes were not so unambiguous. At a high content of Sb_2_S_3_ (**1** and **2**), there were practically no differences in the spectra. At a relatively low content of Sb_2_S_3_ (**4** and **5**), differences in the spectra were observed. It was not possible to unambiguously decompose them into component peaks without fixing the spectral position of these peaks because of the smooth shape of the spectra. Therefore, for a more detailed analysis of the spectra, let us consider the difference between the Raman spectra of glasses with the maximum and minimum Sb_2_S_3_ contents at a constant ratio of Ga_2_S_3_ to GeS_2_ (**1**–**5** and **2**–**4**, two sections). The subtraction was carried out on the unreduced spectra (Figure 7A).

If there were no influence of the structural environment on the SbS_3/2_ pyramid, then we should have observed the spectrum of glassy Sb_2_S_3_ in Figure 7A. Indeed, despite the fact that these two spectra belong to different sections, they were very similar. The spectral position of the maximum of the main peak (297 cm^−1^) corresponded to the spectral position of the fully symmetrical vibration of the SbS_3/2_ pyramid that was assumed in the literature. Variations of the intensity in the 350–450 cm^−1^ range, apparently, were due to the influence of the structural environment. Moreover, this effect increased with an increase in the relative content of Ga_2_S_3_.

The Raman spectrum of the glasses with a constant minimum content of Sb_2_S_3_ (**4** and **5**) is shown in Figure 7B. By subtracting the spectrum of glass **5** from the spectrum of glass **4**, we should obtain the spectrum of the hypothetical glassy Ga_2_S_3_ and the dip corresponding to the spectrum of GeS_2_, which was compensated based on the spectrum of glassy GeS_2_. In the resulting spectrum, the most intense mode was observed at ~320 cm^−1^. It agreed with the data from Reference [50], in which it was assumed that the fully symmetrical vibrations of the GaS_4/2_^−^ tetrahedral structural unit corresponded to the 320 cm^−1^ band.

The second most intense mode was located at about 270 cm^−1^. This band was thought to be due to the presence of non-stoichiometric Ga-Ga bonds (260 cm^−1^) [50]. The low-intensity mode in the region of 420 cm^−1^ could be attributed to the GaS_4/2_ edge-connected tetrahedra, by analogy, with GeS_2_. Thus, it can be assumed that the spectrum shown in Figure 7B corresponded to the hypothetical glassy Ga_2_S_3_.

For the glasses with a constant maximum content of Sb_2_S_3_ (**1** and **2**), this approach was not informative, since the spectra of glasses **1** and **2** were very similar (see Figure 6). Apparently, this was due to the large scattering cross-section of Sb_2_S_3_. However, from a comparison of spectra **1** and **2** in Figure 6, we can conclude that with an increase in the Ga_2_S_3_ content, the intensity of the 250 cm^−1^ signal increased. The latter, with a certain assumption, could be explained by an increase in the part of Ga-Ga homobonds.

**Figure 8:**
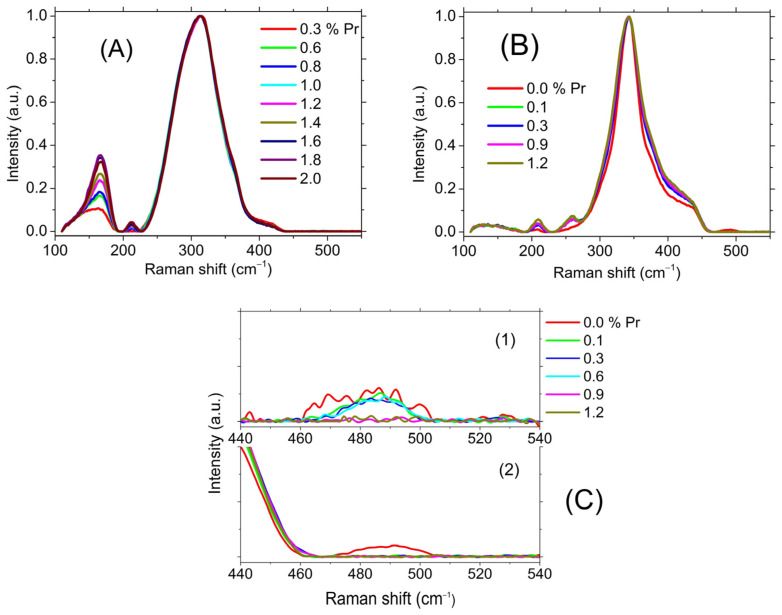
The Raman spectra of the studied glasses containing Pr^3+^ ions. (**A**) composition **1**; (**B**) composition **5**; (**C**) the enlarged scale of the spectra in the region of the peak due to vibrations of S-S bonds ((1) is composition **2**; (2) is composition **5**).

Now, let us consider the effect of the introduction of the REIs on the structure of the studied glasses. The Raman spectra of two studied glassy matrices—e.g., with different contents of Pr^3+^ ions—are shown in Figure 8.

The introduction of a rare earth ion into the glasses with a minimum relative content of Ga_2_S_3_ (**1** and **5**) had the greatest effect on the spectrum. The most pronounced effect was observed for glass with a maximum relative content of Sb_2_S_3_ (**1**). The main changes in the Raman spectra of all studied compositions were the following: peak at 485 cm^−1^ disappeared; peaks at about 170, 210, and 260 cm^−1^ appeared (Table S3, Supplementary Materials).

The question arises about the identification of the bands appearing at about 170 and 210 cm^−1^ upon the introduction of the Pr. The Raman signals at these frequencies were observed in lanthanide sulfides—e.g., the band at 220 cm^−1^ was associated with vibrations of the La–S [56] or Tm–S [57] bonds. However, the concentration of rare earth ions is so low that it is unlikely that these bands were caused by Pr-S bond vibrations.

On the other hand, the characteristic coordination of Pr^3+^ ions by sulfur atoms was no less than 6 (the coordination of La^3+^ and Ce^3+^ ions in chalcogenide glasses is 8) [58]; therefore, the introduction of Pr into the glass matrix should have led to a deficiency of sulfur atoms relative to the stoichiometric composition. If we look at the Raman spectrum—e.g., of **5** in the region of the band caused by vibrations of S-S bonds (~485 cm^−1^) on an enlarged scale (Figure 8C)—then we will see that non-stoichiometric S-S bonds in the original matrix disappeared already with the introduction of 0.1 at.% Pr^3+^ ions.

It should be noted that the replacement of GeS_2_ by Ga_2_S_3_ reduced this effect. Thus, for **2**, non-stoichiometric S-S bonds disappeared when 0.9 at.% of Pr^3+^ ions were introduced (Figure 8C). Thus, a deficiency of sulfur atoms was formed, leading to the formation of metal–metal (M-M) homobonds. Indeed, vibrations of Sb-Sb bonds corresponded to a band at 163–170 cm^−1^ [59,60]. At the same time, the band at 210 cm^−1^ could be attributed to vibrations of mixed Sb–Ge bonds [61,62]. This attribution corresponded to a change in the intensity of these bands with a change in the composition of the glassy matrix. The maximum intensity of these bands was observed for glasses with the maximum relative content of Sb_2_S_3_ and GeS_2_ (**1**). The effect of an increase in the fraction of M–M bonds at the addition of Pr was consistent with the shift of the fundamental absorption edge to the long wavelength range of the spectrum. In Ga_2_S_3_, according to the stoichiometry, there was not enough sulfur to form the structural unit GaS_4/2_, which is characteristic of glasses containing Ga_2_S_3_. As a result, Ga-Ga bonds and three-coordinated sulfur appeared in the glass. The proportion of the three-coordinated sulfur increased with a decreasing sulfur–gallium ratio in the sample. Thus, almost all sulfur had a coordination of three in the amorphous GaS film [63]. Therefore, the absence of a sharp increase in the proportion of M–M bonds in glasses rich in Ga_2_S_3_ upon the introduction of Pr was apparently due to the fact that gallium promotes an increase of sulfur coordination from two to three.

## 4. Conclusions

Pr^3+^ ions were non-uniformly distributed in the Ga_2_S_3_-GeS_2_-Sb_2_S_3_ glass matrix that led to a dipole–dipole interaction between ions and strong concentration quenching. The degree of non-uniformity of distribution increased with an increase in the relative content of Sb_2_S_3_. For compositions containing 65 mol.% Sb_2_S_3_, the luminescence intensity decreased for Pr concentrations of more than 0.1 at.%. With a decrease in the content of Sb_2_S_3_, it was possible to effectively introduce Pr into glasses up to 0.3 at.%. Complex structural units of GaS_4/2_^+^ effectively contributed to the dissolution of REIs in the glass matrix, only up to a certain limit of Ga_2_S_3_ concentrations. Thus, for compositions containing 13 and 35 mol.% Ga_2_S_3_, the concentration positions of the maximum luminescence intensity were approximately the same, which was apparently due to the saturation of the REI surroundings with the GaS_4/2_**^+^** structural units. The calculations based on the Judd–Ofelt theory, which does not take into account the nature of REI distribution, showed that, namely, glasses with a high content of Sb_2_S_3_ should have a high luminescence efficiency. Moreover, at the same Sb_2_S_3_ content, glasses with a higher Ga_2_S_3_ content should have a higher luminescence efficiency. In addition, the studied glasses with an Sb_2_S_3_ content of 65 mol % did not crystallize at a heating rate of 10 K/min; therefore, they could be considered as materials for not only fiber optics, but also for the fabrication of bulk optical elements using an intensively developing molding technique. If it would be possible to reduce the degree of inhomogeneity of the REI distribution, for example, by optimizing the temperature of quenching and increasing the quenching rate, then the glasses with a high content of Sb_2_S_3_ were the most technologically advanced and promising as IR luminescent materials.

The Raman spectroscopy data for a wide range of compositions made it possible to identify the Raman spectra of hypothetical bulk glassy Sb_2_S_3_ and Ga_2_S_3_. The obtained spectrum of the hypothetical bulk glassy Ga_2_S_3_ demonstrated a large fraction of nonstoichiometric Ga-Ga bonds. Adding a few tenths of a percent of Pr to glasses led to a very significant increase in the content of Sb-Sb, Sb-Ge, and Ge-Ge bonds, which probably were in the third coordination sphere of REI. The magnitude of this effect increased with an increase in the proportion of Sb_2_S_3_ and GeS_2_ in the glass. Reducing this effect for glasses enriched with Ga_2_S_3_ could be explained by the assumption that gallium promotes the formation of three-coordinated sulfur, which reduces the deficit of chalcogen and reduces the fraction of M-M bonds.

## Figures and Tables

**Table 1:** Composition and physicochemical properties of the glasses with the compositions **1**–**5**. The refractive index is specified for a wavelength of 1.2 µm.

No Composition	GaS_1.5_ (mol. %)	GeS_2_ (mol. %)	SbS_1.5_ (mol. %)	d (g/cm^3^)	n	T_g_ (°C)	T_cr_ (°C)
1	5.59	29.41	65.00	3.92	2.4	258	absent
2	14.71	20.29	65.00	3.85	2.5	240	absent
3	30.01	41.39	28.60	3.42	2.4	312	400
4	35.03	48.31	16.66	3.34	2.3	354	414
5	13.31	70.03	16.66	3.28	2.2	346	483

**Table 2:** Spectral position of the luminescence bands in the studied glasses upon excitation at 607 nm (^3^H_4_-^1^D_2_) and their identification.

No	Wavelength (μm)	Energy (cm^−1^)	Transition
1	0.833	12,005	^1^D_2_-^3^H_6_
2	0.867	11,534	^1^D_2_-^3^F_2_
3	0.9	11,111	Nd^3+^ (^4^F_3/2_-^4^I_9/2_)
4	1.01	9901	^1^D_2_-^3^F_3_
5	1.041	9606	^1^D_2_-^3^F_4_ and ^1^G_4_-^3^H_4_
6	1.081	9251	Nd^3+^ (^4^F_3/2_-^4^I_11/2_)
7	1.336	7485	^1^G_4_-^3^H_5_

**Table 3:** The Judd–Ofelt parameters for the glasses of compositions **1**–**5** containing Pr^3+^ ions.

No Composition	Ω_2_ (10^−20^ cm^2^)	Ω_4_ (10^−20^ cm^2^)	Ω_6_ (10^−20^ cm^2^)	Radiative Lifetime from ^1^G_4_ (ms)
1	9.77	5.34	5.38	0.304
2	8.13	5.39	4.78	0.287
3	10.04	2.36	5.67	0.330
4	9.73	4.45	5.69	0.361
5	12.62	2.95	6.66	0.385

**Table 4:** The calculated transition probabilities for the studied glasses of compositions **1**–**5**.

Transition	Transition Probabilities (s^−1^)				
	1	2	3	4	5
^1^G_4_-^3^F_4_	117.2	124.6	106.6	98.4	91.4
^1^G_4_-^3^F_3_	17.9	18.8	17.8	15.6	15.1
^1^G_4_-^3^F_2_	16.7	19.1	10.4	12.7	9.0
^1^G_4_-^3^H_6_	1110.5	1168.0	975.9	909.4	855.8
^1^G_4_-^3^H_5_	1804.5	1917.2	1709.1	1545.1	1449.7
^1^G_4_-^3^H_4_	224.4	240.2	208.8	191.6	176.6

## Data Availability

Not applicable.

## Supplementary Materials

The following supporting information can be downloaded at: https://www.mdpi.com/article/10.3390/ma16134672/s1. Refs. [64,65] are cited in the Supplementary Materials.
