# Supplementary material for: Structure and Luminescent Properties of Glasses in the GeS2-Ga2S3-Sb2S3:Pr3+ System
Source: Materials (Basel). 2023 Jun 28;16(13):4672. doi: 10.3390/ma16134672 (PMC10342960; doi:10.3390/ma16134672)
Supplement: Supplementary file 1 [file materials-16-04672-s001.zip › materials-2433553-supplementary.pdf]

Supplementary

1. The examples of X-ray diffraction pattern and microscope image of the synthesized glasses.

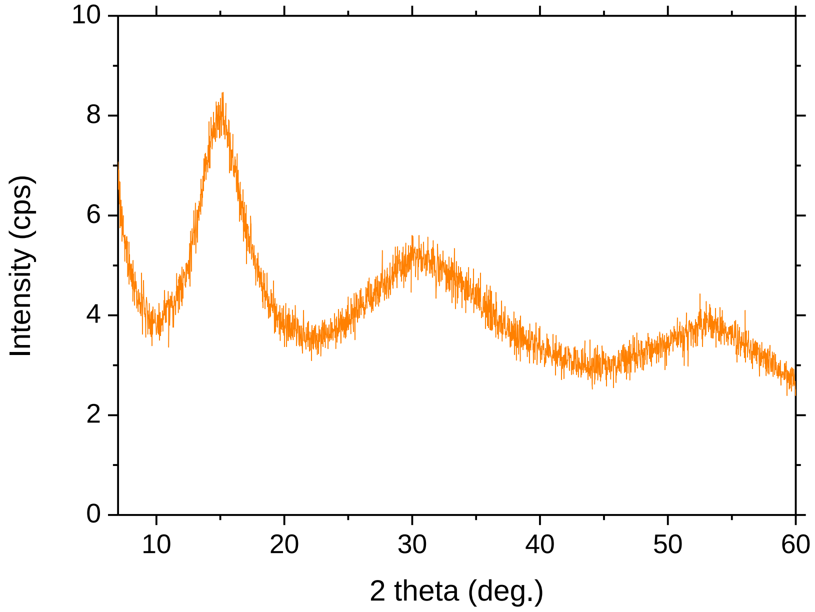

(a)

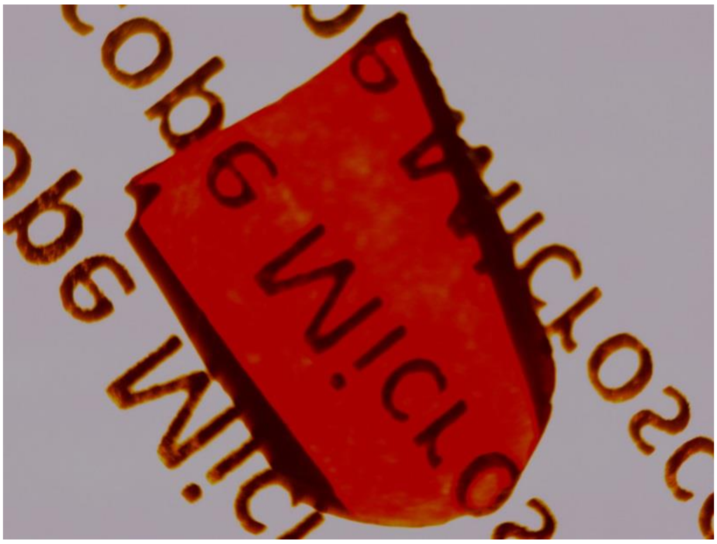

(b)

Figure S1. (a) X-ray diffraction pattern of the glass with composition **5**. (**b**) Microscope image of the glass with composition **3** with 0.1 at. % Pr placed on paper with the printed text.

2. **Table S1**. Spectral position of the absorption bands of the studied glasses caused by the transitions of Pr^{3+} ions, the energy of the transitions, and their identification.

Peak	Wavelength, nm	Energy, cm^{-1}	Transition
1	602	16611,3	$^3H_4$-$^1D_2$
2	1026	9746,6	$^3H_4$-$^1G_4$
3	1478	6765,9	$^3H_4$-$^3F_4$
4	1582	6321,1	$^3H_4$-$^3F_3$
5	2020	4950,4	$^3H_4$-$^3F_2$
6	2347	4260,8	$^3H_4$-$^3H_6$
7	4738	2110,6	$^3H_4$-$^3H_5$

3. Two peaks in the luminescence spectrum (see Figure 4 in the manuscript) are observed in the region of 0.9 and 1.081 μm, which do not agree with the level diagram of the Pr^{3+} ion. It can be argued that they can be referred to as the admixture of Nd^{3+} ions—namely, the transitions from the $^4F_{3/2}$ to the $^4I_{9/2}$ and $^4I_{11/2}$ levels of the Nd^{3+} ion. This happens, firstly, since Pr contains a significant proportion of Nd impurities and, secondly, the used excitation radiation with a wavelength of 607 nm should also pump the $^2G_{7/2}$ and $^4G_{5/2}$ levels of the Nd^{3+} ion. The association of these two peaks with the impurity Nd^{3+} was assumed in Reference [S1]. This relationship can be seen more clearly after an analysis of the Raman spectra of the glasses with Pr^{3+} ions. The Raman spectrum of the glass with composition **2** with 0.3 at. % Pr^{3+} upon excitation by a laser with a wavelength of 785 nm is shown in Figure S1. Three peaks are visible on the spectrum in the range between 1300 and 2000 cm^{-1}, which cannot be referred to as the Raman effect.

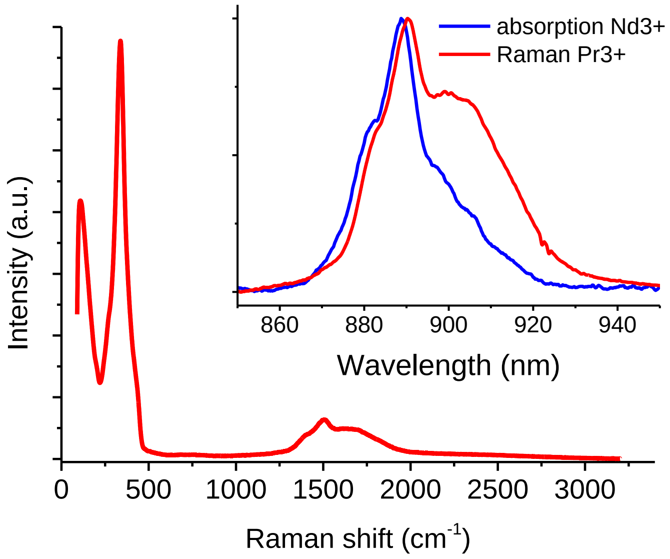

Figure S2. The Raman spectrum of **2** with 0.3 at. % Pr^{3+}. Inset—the part of the Raman spectrum of the glass and the absorption spectrum of the chalcogenide glass with Nd^{3+} in the same spectral range.

At the same time, if this range of the Raman spectrum is rearranged in the intensity–wavelength coordinates and compared with the absorption spectrum of the chalcogenide glass with Nd^{3+} ions, then their relationship is clearly visible (Figure S2, inset)*. Thus, when the Raman signal is initiated by a laser light with a wavelength of 785 nm, the Nd^{3+} luminescence is also excited. The latter confirms the presence of an Nd impurity in Pr and consistent with the assignment of two peaks in the luminescence spectra of the obtained glasses to the luminescence of Nd^{3+} ions.

* - Transformation of the Raman spectrum from the energy scale (cm^{-1}) to the wavelength scale (nm) was carried out, taking into account the Jacobian intensity correction [S2].

S1. Quimby R.S; Aitken B.G. Anomalous temperature quenching of fluorescence in Pr^{3+} doped sulfide glass. *J. Appl. Phys*. **1997**, 82 (8), 3992-3996.

S2. Mooney J.; Kambhampati P. Get the basics right: Jacobian conversion of wavelength and energy scales for quantitative analysis of emission spectra. *J. Phys. Chem. Lett*. **2013**, 4 (19), 3316-3318. DOI: 10.1021/jz401508t.

4. **Table S2**. The calculated values of the branching ratio in the studied glasses.

1 – **5** are the numbers of the samples with different compositions.

transition	Branching ratio				
	**1**	**2**	**3**	**4**	**5**
$^1G_4$-$^3F_4$	3.56E-2	3.57E-2	3.52E-2	3.55E-2	3.52E-2
$^1G_4$-$^3F_3$	5.44E-3	5.40E-3	5.88E-3	5.63E-3	5.80E-3
$^1G_4$-$^3F_2$	5.06E-3	5.47E-3	3.43E-3	4.57E-3	3.46E-3
$^1G_4$-$^3H_6$	0.337	0.335	0.322	0.328	0.329
$^1G_4$-$^3H_5$	0.546	0.550	0.564	0.557	0.558
$^1G_4$-$^3H_4$	6.82E-2	6.89E-2	6.90E-2	6.91E-2	6.89E-2

5. **Table S3**. Changes in the Raman spectra after the introduction of Pr^{3+} into the glasses.

Sample #	Changes in the Raman spectrum
**1**	peaks appear at 166 and 213 cm^{-1}
**2**	peak appears at 166 cm^{-1}; peak disappears at 485 cm^{-1}
**3**	peaks appear at 170 and 210 cm^{-1}
**4**	peaks appear at 170 and 208 cm^{-1}
**5**	peaks appear at 208 and 260 cm^{-1}; peak disappears at 485 cm^{-1}
